# Trehalose Alleviates D-Galactose-Induced Aging-Related Granulosa Cell Death in Ovaries

**DOI:** 10.3390/ijms252312643

**Published:** 2024-11-25

**Authors:** Huaming Xi, Xinyu Chen, Kai Liang, Xianglong Wang, Feng Jiang, Yuan Li, Dong Niu

**Affiliations:** Key Laboratory of Applied Technology on Green-Eco-Healthy Animal Husbandry of Zhejiang Province, Zhejiang Provincial Engineering Laboratory for Animal Health Inspection & Internet Technology, Zhejiang International Science and Technology Cooperation Base for Veterinary Medicine and Health Management, China-Australia Joint Laboratory for Animal Health Big Data Analytics, College of Animal Science and Technology & College of Veterinary Medicine of Zhejiang A&F University, Hangzhou 311300, China; xihuaming@zafu.edu.cn (H.X.); chenxinyu@stu.zafu.edu.cn (X.C.); 202202150226@stu.zafu.edu.cn (K.L.); wangxl@stu.zafu.edu.cn (X.W.); jiangf@stu.zafu.edu.cn (F.J.)

**Keywords:** trehalose, granulosa cell, aging, mitophagy, ovary

## Abstract

Ovarian dysfunction caused by aging restricts female reproductive capacity and is accompanied by oxidative stress and impaired autophagy. Recent studies have shown that trehalose (Tre) can activate autophagy and have antioxidant effects. However, whether Tre can be used to attenuate ovarian aging remains unclear. Therefore, the anti-aging effects of Tre on the ovary were explored both in vivo and in vitro. D-galactose (D-gal) was administered i.p. daily (200 mg/kg body weight) for 8 weeks to establish the mouse ovarian aging model (n = 10). We found that Tre significantly reversed ovarian weight loss and reduced the number of TUNEL-positive granulosa cells caused by D-gal in mouse ovaries. Tre elevated the protein expression levels of LC3-II, Parkin, PINK1, Beclin1, and LAMP2 in ovaries. Mitochondrial-related proteins TOM20 and COX IV expression levels were increased by Tre administration. In vitro studies further supported these findings, showing that Tre treatment significantly reduced the number of SA-β-gal and PI-positive cells, and decreased ROS levels in cultured granulosa cells. Thus, Tre alleviates ovarian aging by activating mitophagy and reducing oxidative stress, suggesting its potential as an anti-aging agent for ovarian health.

## 1. Introduction

Aging leads to impaired organ function, accompanied by the degeneration of cells and tissues [1]. Women’s fertility begins to decline at the age of 35, and reproductive function stops completely at menopause [2]. During ovarian aging, the quality of oocytes decreases, and the number of follicles decreases, ultimately leading to loss of fertility [3]. Ovarian aging is mainly caused by excessive oxidative stress in the ovarian microenvironment, closely related to follicular cells, hormones, and growth factors [4]. However, the ovarian aging process is regulated by multiple factors [5,6,7]. The follicle is mainly composed of granulosa cells and oocytes. Granulosa cells in mature follicles secrete large amounts of estrogen and growth factors, which play a key role in the development and maturation of oocytes [8]. Signal molecules secreted by oocytes induce the proliferation and differentiation of granulosa cells, creating cross-talk and interaction between them. Granulosa cells play a pivotal role in the development and maturation of oocytes. They are also essential for maintaining the normal endocrine environment necessary for oocyte health. During the aging process, the level of estrogen secreted by granulosa cells decreases [9], further aggravating ovarian damage.

Under normal conditions, reactive oxygen species (ROS) content is crucial to the process of follicle development, oocyte maturation, fertilization, and embryonic development [10]. However, excessive ROS can cause ovarian oxidative stress, inhibiting follicle development and reducing oocyte quality. Oxidative stress causes ovarian dysfunction [11], but granulosa cells can protect oocytes from damage by ROS within the follicle. ROS accumulation caused by aging is the main cause of granulosa cell apoptosis [12], which can lead to damage to the ovarian microenvironment and nutrient deficiency in oocytes, resulting in impaired ovarian function [13]. Moreover, apoptotic cells increase the cell-free DNA content in follicular fluid and induce the massive production of ROS, which further exacerbates human ovarian cell apoptosis [14]. Many studies have shown that aging-induced oxidative stress leads to mitochondrial dysfunction accompanied by impaired autophagy [15,16,17].

Autophagy, as an important mechanism for maintaining cell homeostasis, can clear damaged proteins and organelles. In the ovaries of aged rats, the expression levels of autophagy-related genes Beclin1 and LC3 are reduced [18], indicating that ovarian aging is associated with a decrease in autophagy levels. Zhang et al. [19] reported that spermidine supplementation can promote follicular development and female fertility in aged mice by activating mitophagy and improving mitochondrial function. Another study also showed that long-term administration of Nicotinamide mononucleotide elevates the autophagy level and increases mitochondrial biogenesis in ovarian granulosa cells in aged mice [20], thereby exerting an anti-aging effect on the ovary. These studies suggest that activating autophagy in ovarian cells to improve mitochondrial function may be involved in resisting ovarian aging.

Trehalose (Tre) is a naturally occurring disaccharide that is used as a bioprotectant to protect cells from various stress conditions [21]. Tre is approved as a safe food for humans by the European regulation system and the U.S. Food and Drug Administration [22]. Moreover, Tre is non-toxic and will not cause rapid changes in blood sugar [22]. In recent years, supplementation with antioxidants, vitamins, and phytochemicals has been used to resist aging, improve ovarian function, and enhance reproductive capacity in humans and mice. Previous studies have shown that antioxidants can clear excess ROS and promote follicle development [23]. Tre has been shown to have an antioxidant role both in vivo and in vitro [24,25,26]. Further study has shown that Tre protects cells from oxidative stress by activating autophagy [27]. Rusmini et al. [28] reported that Tre induces autophagy by promoting lysosomal biogenesis. Furthermore, Tre-activated autophagy alleviates stress-induced mitochondrial dysfunction [29], and inhibits cell apoptosis [30]. Administration of Tre has also shown excellent anti-aging effects on age-associated cognitive impairment and brain aging of rats [31] and mice [32]. Impaired autophagy is one of the hallmarks of organ aging [33]. Tre, as an autophagy activator, can promote lysosomal biogenesis and promote the autophagic degradation process. However, it remains unclear whether trehalose can activate follicular cell autophagy and thereby alleviate oxidative stress and promote follicular cell survival to resist ovarian aging. The objective of the present study was to explore the effect of Tre on ovarian aging in a D-gal-induced mouse aging model.

## 2. Results

### 2.1. Tre Attenuates D-Gal-Induced Apoptosis and Oxidative Stress in Granulosa Cells

**Figure 1:**
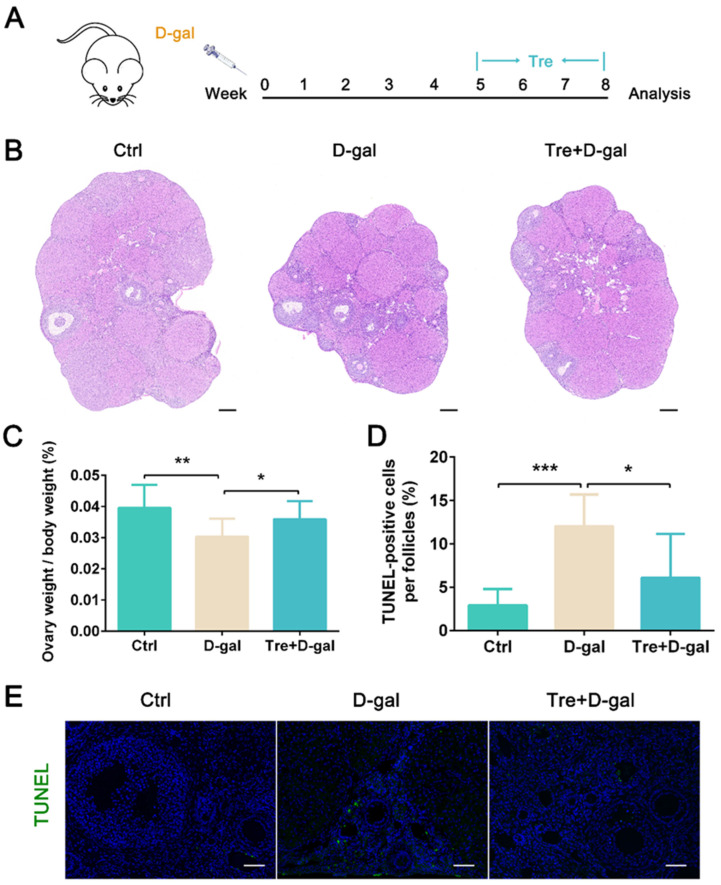
Tre attenuates D-gal-induced apoptosis in granulosa cells. (**A**) Female mice were treated with D-gal (200 mg/kg body weight), Tre (2 g/kg body weight), or normal saline. (**B**) Histological changes in ovaries were examined by hematoxylin and eosin staining. Bar = 200 μm. (**C**) Quantification analysis of ovary weight/body weight. n = 10. (**D**) Quantification analysis of TUNEL-positive cells per follicles. (**E**) Representative images of TUNEL staining. Bar = 50 μm. * *p* < 0.05, ** *p* < 0.01, *** *p* < 0.001. Ctrl, control group. D-gal, D-gal group. Tre+D-gal, Tre+D-gal group.

**Figure 2:**
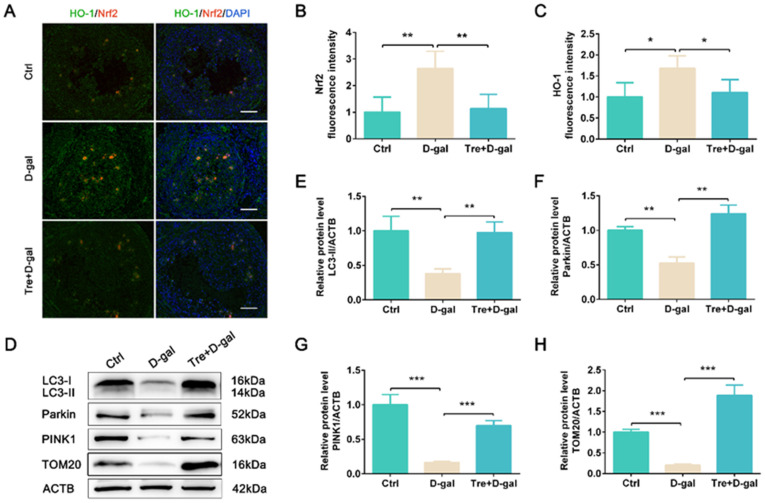
Tre attenuates oxidative stress induced by D-gal in mouse ovaries. (**A**) Representative images of fluorescence-labeled Nrf2 and HO-1. Bar = 50 μm. (**B**,**C**) Fluorescence intensity quantification of Nrf2 and HO-1. (**D**–**H**) The expression levels of LC3, Parkin, PINK1, and TOM20 were detected by Western blot. n = 6. * *p* < 0.05, ** *p* < 0.01, *** *p* < 0.001. Ctrl, control group. D-gal, D-gal group. Tre+D-gal, Tre+D-gal group.

To explore the protective effect of Tre against ovarian aging, D-gal was used to establish a mouse ovarian aging model (Figure 1A). The ovarian tissues were stained with hematoxylin and eosin, and the results shown that D-gal treatment reduced ovarian size. Administration of Tre significantly (*p* < 0.05) increased ovarian weight compared to the D-gal treatment group (Figure 1B,C). To clarify whether the reduction in ovarian morphology is related to cell apoptosis, we detected the number of apoptotic cells in ovarian tissue. We found that Tre significantly (*p* < 0.05) reduced the number of TUNEL-positive granulosa cells induced by D-gal treatment (Figure 1D,E). In addition, D-gal-induced ovarian aging is accompanied by oxidative stress; Tre treatment significantly (*p* < 0.05) reduced the fluorescence intensity of Nrf2 and HO-1 (Figure 2A–C), indicating that Tre alleviated ovarian oxidative stress.

### 2.2. Tre Activates Mitophagy and Promotes Mitochondrial Biogenesis

**Figure 3:**
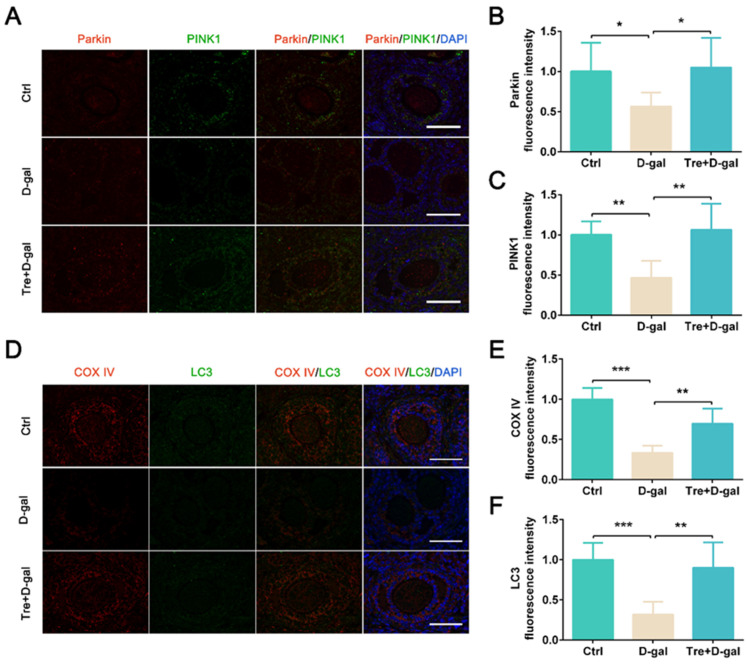
Tre activates mitophagy in D-gal-treated ovaries. (**A**) Representative images of fluorescence-labeled Parkin and PINK1. Bar = 50 μm. (**B**,**C**) Fluorescence intensity quantification of Parkin and PINK1. (**D**) Representative images of fluorescence-labeled LC3 and COX IV. Bar = 50 μm. (**E**,**F**) Fluorescence intensity quantification of LC3 and COX IV. * *p* < 0.05, ** *p* < 0.01, *** *p* < 0.001. Ctrl, control group. D-gal, D-gal group. Tre+D-gal, Tre+D-gal group.

To determine whether Tre can activate mitophagy in D-gal-induced ovarian aging, the expression of mitophagy-related proteins was analyzed. The results of Western blot show that Tre administration significantly increased the expression levels of LC3-II (*p* < 0.01), Parkin (*p* < 0.01), PINK1 (*p* < 0.001), and TOM20 (*p* < 0.001) compared to the D-gal treatment group (Figure 2D–H). Furthermore, IF staining shows the fluorescence intensity of Parkin and PINK1 was reduced (*p* < 0.05) by treatment with Tre in follicles of the Tre+D-gal group (Figure 3A–C). The association of mitochondria with autophagosomes indicates the activation of mitophagy. Tre treatment significantly increased the co-localized fluorescence intensity of LC3 and COX IV in granulosa cells (Figure 3D–F). The results suggested that Tre can activate the mitophagy of granulosa cells.

### 2.3. Tre Promotes Autophagic Flux in Granulosa Cells

**Figure 4:**
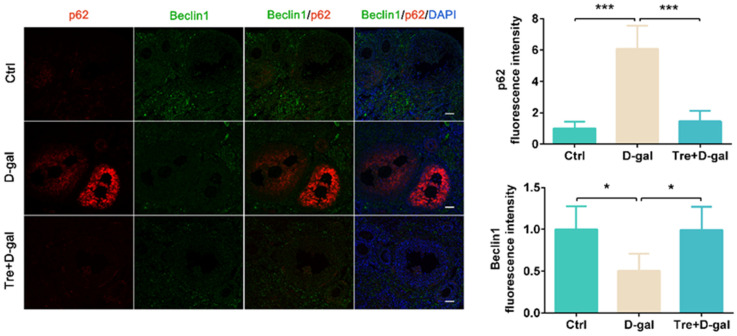
Tre promotes autophagic flux in granulosa cells of ovaries. Immunofluorescence of p62 and Beclin1 in Ctrl, D-gal, Tre+D-gal groups. Bar = 50 μm. * *p* < 0.05, *** *p* < 0.001. Ctrl, control group. D-gal, D-gal group. Tre+D-gal, Tre+D-gal group.

**Figure 5:**
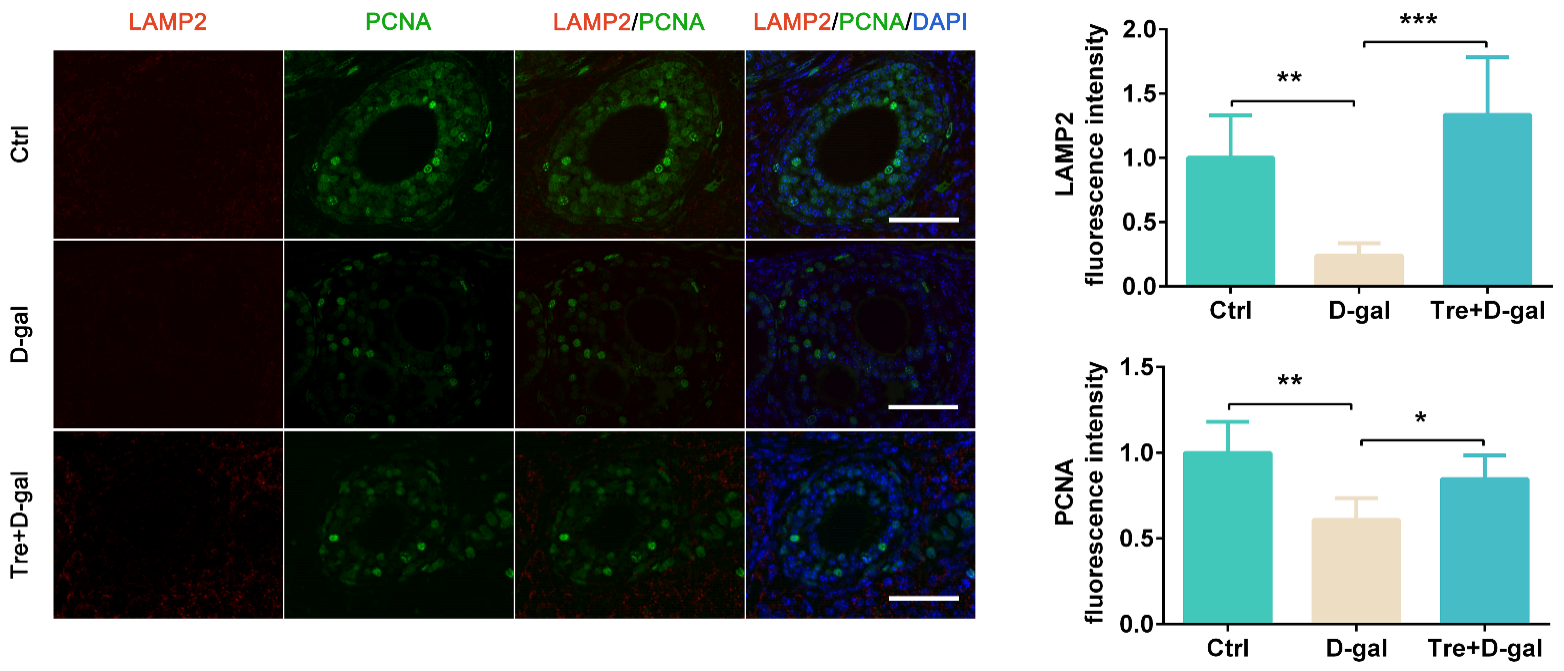
Tre induces lysosomal biogenesis in granulosa cells of ovaries. Immunofluorescence of LAMP2 and PCNA in Ctrl, D-gal, Tre+D-gal groups. Bar = 50 μm. * *p* < 0.05, ** *p* < 0.01, *** *p* < 0.001. Ctrl, control group. D-gal, D-gal group. Tre+D-gal, Tre+D-gal group.

We analyzed the effects of D-gal and Tre on autophagic flux by immunofluorescence staining. The results shown that expression levels of Beclin1 and LAMP2 were significantly (*p* < 0.05) higher in granulosa cells of the Tre+D-gal group than in the D-gal group (Figure 4 and Figure 5). Tre treatment significantly (*p* < 0.001) reduced p62 fluorescence intensity in granulosa cells compared to the D-gal group (Figure 4). In addition, PCNA was localized in granulosa cells and was increased (*p* < 0.05) after treatment with Tre (Figure 5), indicating that Tre improved the proliferation activity of granulosa cells.

### 2.4. Tre Alleviates D-Gal-Induced Granulosa Cell Death In Vitro

**Figure 6:**
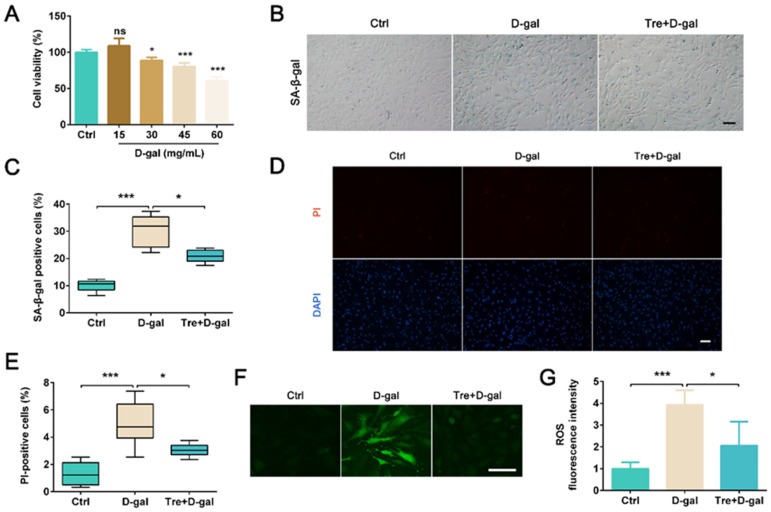
Tre alleviates D-gal-induced granulosa cell death in vitro. (**A**) Granulosa cells were isolated and stimulated with different concentrations of D-gal (15, 30, 45, and 60 mg/mL) for 24 h. CCK-8 assay was performed to detect the granulosa cell viability. n = 5. (**B**,**C**) Granulosa cells were pretreated with 10 mmol/L Tre for 24 h, then exposed to 45 mg/mL D-gal for 24 h. Cell senescence was examined using SA-β-gal staining. n = 5. Bar = 100 μm. (**D**) Representative images of fluorescence-labeled PI. Bar = 100 μm. (**E**) Quantification analysis of PI-positive cells. n = 6. (**F**,**G**) ROS levels were determined by DCFH-DA staining in different groups. n = 5. Bar = 100 μm. ns, no significance. * *p* < 0.05, *** *p* < 0.001. Ctrl, control group. D-gal, D-gal group. Tre+D-gal, Tre+D-gal group.

To further clarify whether Tre can protect granulosa cells from D-gal-induced aging-related injury, we isolated mouse granulosa cells and cultured in vitro. Granulosa cells were treated with different concentrations of D-gal (15, 30, 45, 60 mg/mL). The results of CCK-8 shown that 45 and 60 mg/mL D-gal significantly (*p* < 0.001) reduced granulosa cell viability (Figure 6A). Thus 45 mg/mL D-gal was used for subsequent experiments. In the Tre+D-gal group, Tre treatment significantly reduced the number of SA-β-gal positive cells (*p* < 0.05, Figure 6B,C) and the number of granulosa cell death (*p* < 0.05, Figure 6D,E) compared to the D-gal group. Further results shown that the increased ROS levels induced by D-gal were significantly decreased by Tre treatment (*p* < 0.05, Figure 6F,G). These results indicated that Tre can alleviate D-gal-induced granulosa cell death in vitro.

## 3. Discussion

Ovarian aging is an important factor affecting female reproductive capacity. With increasing age, ROS levels in the ovary increase, and antioxidant enzyme activities decrease, which leads to ovarian oxidative stress. In aging-related research, D-gal is widely used to partially simulate physiological senescence in mice and then explore the regulatory mechanism of organ aging. A previous study has shown that D-gal treatment causes ovarian oxidative damage, inhibits granulosa cell function, and blocks ovarian development in mice [34]. Further findings showed that exposure to D-gal led to advanced glycation end-product accumulation in mouse ovaries [35], which exacerbated ovarian aging and functional decline [36]. Consistent with a previous study [34], our study also showed that D-gal reduced ovarian size and increased ovarian cell apoptosis in mice. Thus, this study used the D-gal-induced ovarian oxidative stress model to analyze the protective effect of Trehalose (Tre).

Granulosa cells play a key role in the maintenance of ovarian function and follicular development. During follicular development, granulosa cells continue to proliferate to provide nutrition and support for follicular development and regulate the ovulation process [37]. PCNA plays an important role in the initiation of cell proliferation and is a key indicator reflecting the state of cell proliferation. A previous study has shown that PCNA is specifically expressed in granulosa cells within follicles [38]. Our findings are consistent with those of Fortune et al. [38], in that PCNA is specifically localized in granulosa cells. Furthermore, Tre treatment significantly increased the expression level of PCNA in granulosa cells in mouse ovaries, indicating that Tre promotes the proliferation of granulosa cells. In the present study, D-gal treatment increased cell apoptosis, all of which was improved after administration of Tre. Ovarian aging is associated with excessive oxidative stress, which reduces female reproductive capacity [39]. Mitochondrial dysfunction caused by aging can lead to severe ovarian dysfunction. Under stress conditions, mitochondrial damage further exacerbates oxidative stress and causes granulosa cell dysfunction [40,41,42]. Mitochondria are key sources of ROS, and excessive ROS disrupts mitochondrial function, promotes cytochrome c release, elevates BAX expression, and triggers ovarian cell apoptosis [43,44]. Mo et al. [45] found that Tre alleviates oxidative stress and apoptosis in IPEC-J2 cells caused by heat stress. Many studies have shown that Tre plays an anti-apoptotic role during cryopreservation of granulosa cells, ovarian tissue, and testicular tissue, thereby promoting the survival of frozen–thawed cells [46,47,48]. Gao et al. [49] also reported that Tre inhibits DHEA-induced apoptosis in KGN cells and ovaries of PCOS mice. These findings confirm that Tre attenuated D-gal-induced granulosa cell apoptosis in mouse ovarian follicles.

Currently, Tre is widely studied for its potential in treating aging-related neurodegenerative diseases [27], with its effects largely attributed to its regulation of autophagy. Mitophagy, a key type of autophagy, is a complex cellular process that clears damaged and redundant mitochondria. Oxidative stress and excess ROS can induce mitophagy activity [50]. Numerous studies have reported that Tre can act as an autophagy activator, regulating cell status and function [51,52]. Zhu et al. [29] reported that Tre attenuates cisplatin-induced mitochondrial dysfunction by activating autophagy in mice. Additionally, Tre promotes lysosomal biogenesis, and Tre-activated autophagy inhibits oxidative stress and apoptosis in mouse liver [53]. These studies suggest that Tre’s regulatory effects on mitochondria and oxidative stress are mediated through the activation of the autophagy pathway. Importantly, Gao et al. [49] showed that Tre benefits the ovary, promotes follicle development, and increases estrogen content in mice. These data support Tre’s potential role in mitophagy and propose future experiments to validate this mechanism. The expression levels of PINK1 and Parkin proteins are related to mitophagy activity. Our study found that LC3, Beclin1, PINK1, and Parkin protein expression levels increased in the granulosa cells of Tre+D-gal treated mice compared to those in the D-gal group. Additionally, the expression level of p62 (an autophagy substrate) decreased in granulosa cells of Tre+D-gal treated mice. These results suggested that D-gal treatment caused mitochondrial damage, whereas Tre administration increased mitophagy activity and promoted mitochondrial clearance. Previous studies have also shown that D-gal reduces autophagy activity [54], and that Tre treatment increases mitophagy activity [55], which aligns with our findings. The above studies support our conclusion that Tre activates PINK1/Parkin-mediated mitophagy to alleviate D-gal-induced aging-related granulosa cell death. However, there were some limitations in this study, such as levels of trehalose and its metabolites in animals, treatment duration, and natural aging animal models, which need to be further explored in future studies. Crucially, whether Tre can affect ovarian development and aging by regulating gap junctions and signaling between granulosa cells and oocytes still needs to be further elucidated using a series of experiments and bioinformatic methodology. But the results of this study provide important evidence for Tre’s role in ovarian development and anti-aging.

## 4. Materials and Methods

### 4.1. Animals and Treatment

All animal experimental procedures were approved by the Institutional Animal Care and Use Committee of Zhejiang A&F University, China (ZAFUAC2023005). Thirty 10-week-old female ICR mice were used in this study. With unrestricted access to food and water, all mice were kept in a 12 h/12 h light/dark cycle environment (temperature 22 ± 2 °C, relative humidity 50–60%). These mice were randomly divided into three groups (n = 10): Control, D-gal, and Tre+D-gal groups. D-gal (200 mg/kg body weight, Sigma-Aldrich, G0625, St. Louis, MO, USA) was daily by intraperitoneal injection (i.p.) for 8 weeks [56] in D-gal and Tre+D-gal groups. Tre (563051, J&K Scientific, Beijing, China) was administered i.p. (2 g/kg body weight) daily for 4 weeks in the Tre+D-gal group (Figure 1A). After treatment for 8 weeks, all mice were weighed and euthanized. Ovarian tissues were immediately weighed. The tissues were fixed in Bouin’s solution or frozen in liquid nitrogen (−196 °C).

### 4.2. Histology and TUNEL Assay

After fixing in Bouin’s solution, the tissues were sliced into 5 μm sections. The sections were stained with hematoxylin and eosin according to the previously described [57]. TUNEL staining was performed according to the manufacturer’s instructions (G1501, Servicebio, China) for analyzing follicle cell apoptosis. The nuclear was counterstained with DAPI (1 μg/mL). The images were captured with a fluorescence microscope (CKX53SF, OLYMPUS, Tokyo, Japan).

### 4.3. Immunofluorescence Staining

For immunofluorescence staining, Bouin’s fixed ovarian sections were sliced into 5 μm sections. The sections were deparaffinized and rehydrated. Then, the sections were incubated with 5% bovine serum albumin for 30 min to block the nonspecific binding sites, stained with primary antibodies overnight at 4 °C, and then exposed to secondary antibody IgG Cy3/FITC (1:500, bs-0296G-Cy3/FITC, BIOSS, Beijing, China) or IgG Cy3/FITC (1:500, bs-0295G-Cy3/FITC, BIOSS, Beijing, China). The primary antibodies included HO-1 (1:400, GB12104, Servicebio, Wuhan, China), Nrf2 (1:300, 16396-1-AP, Proteintech, Rosemont, IL, USA), PINK1 (1:300, GB114934, Servicebio, Wuhan, China), Parkin (1:200, 66674-1-Ig, Proteintech, Rosemont, IL, USA), LC3 (1:100, A19665, Abclonal, Wuhan, China), COX IV (1:500, GB12250, Servicebio, Wuhan, China), Beclin1 (1:300, 66665-1-Ig, Proteintech, Rosemont, IL, USA), p62 (1:500, 18420-1-AP, Proteintech, Rosemont, IL, USA), PCNA (1:500, GB11010, Servicebio, Wuhan, China), and LAMP2 (1:400, GB11330, Servicebio, Wuhan, China). DNA was counterstained with DAPI (1 μg/mL). Immunofluorescence staining was examined using a fluorescence microscope (CKX53SF, OLYMPUS, Tokyo, Japan).

### 4.4. Western Blot

The ovarian tissues were lysed using RIPA (P0013B, Beyotime, Shanghai, China) containing PMSF (ST507, Beyotime, Shanghai, China). Protein lysates (20 μg of total proteins) were separated on 10–12% SDS-PAGE and transferred onto NC membranes (Millipore, MA, USA). The membranes were blocked with 5% nonfat milk (1 h, room temperature). The membranes were incubated with primary antibodies overnight at 4 °C, followed by secondary antibody staining (1:10,000, CW0102S and CW0103S, CWBIO, Beijing, China) for 1 h (37 °C). The primary antibodies included LC3 (1:1000, A19665, Abclonal, China), TOM20 (1:3000, A19403, Abclonal, China), Parkin (1:500, sc-32282, Santa Cruz, CA, USA), PINK1 (1:600, 23274-1-AP, Proteintech, USA), and ACTB (1:5000, 20536-1-AP, Proteintech, USA). Proteins were visualized with ECL Enhanced Kit (RM00021, Abclonal, Wuhan, China), and quantified by ImageJ 1.53t software.

### 4.5. Isolation and Culture of Follicular Cells

For isolation of granulosa cells, 10-week-old female ICR mice were injected with 10 IU of pregnant mare serum gonadotropin (PMSG) for 48 h. Then, mice were euthanized to collect these ovaries. The pre-ovulatory follicles on the ovarian surface were punctured to collect granulosa cells under a stereomicroscope. The obtained granulosa cells were washed and cultured in Dulbecco’s modified eagle’s medium F-12 (DMEM/F12 with 15 mM HEPES, pH 7.4) containing 10% fetal bovine serum (FBS, 100–123, GEMINI, West Sacramento, CA, USA), 100 IU/mL penicillin, and 100 μg/mL streptomycin (Invitrogen, Carlsbad, CA, USA) at 37 °C in 5% CO_2_. The following experiments were performed with first-passage granulosa cells.

### 4.6. Cell Treatment

Granulosa cells were stimulated with D-gal (15, 30, 45, 60 mg/mL) for 24 h to construct an in vitro aging injury model. For the group treated with D-gal (G0625, Sigma-Aldrich, MO, USA) and Tre (563051, J&K Scientific, Beijing, China), granulosa cells were pretreated with or without Tre (10 mmol/L) for 24 h, and then exposed to 45 mg/mL D-gal for 24 h.

### 4.7. Cell Viability

CCK-8 assay was used to detect granulosa cell viability. Granulosa cells were seeded in 96-well plates (3000 cells per well) and then exposed to the designed treatment. Then, 10 μL CCK-8 reagent (C0005, TargetMol, Shanghai, China) was added to each well and incubated for 3 h in the dark. Absorbance at 450 nm was measured using a microplate reader (iMark, Bio-Rad, Hercules, CA, USA).

### 4.8. SA-β-Gal Staining

After exposure, granulosa cells were fixed with 4% paraformaldehyde for 15 min. Then, cells were stained with a cell senescence β-galactosidase staining kit (C0602, Beyotime, Shanghai, China). The images were collected, and SA-β-gal-positive cells were counted.

### 4.9. Cell Death Assay

For the cell death assay, granulosa cells were incubated with Propidium Iodide (PI, 10 μmol/L, C0080, Solarbio, Beijing, China). An amount of 10 μL PI solution was added to each well and incubated for 20 min. The nucleus was counterstained with DAPI (1 μg/mL). Images were collected using a fluorescence microscope (CKX53SF, OLYMPUS, Tokyo, Japan).

### 4.10. ROS Levels

ROS assay kit (S0033S, Beyotime, Shanghai, China) was used to detect the ROS levels in granulosa cells according to the manufacturer’s instructions. Briefly, granulosa cells were incubated with 10 μmol/L DCFH-DA for 30 min at 37 °C. Then, cells were scanned under a fluorescence microscope (CKX53SF, OLYMPUS, Tokyo, Japan).

### 4.11. Statistical Analysis

All data were analyzed using GraphPad Prism software (version 6). Statistical analysis was done by one-way analysis of variance (ANOVA). All data were represented as mean ± standard error of the mean (SEM). *p* < 0.05 is considered as significant.

## 5. Conclusions

**Figure 7:**
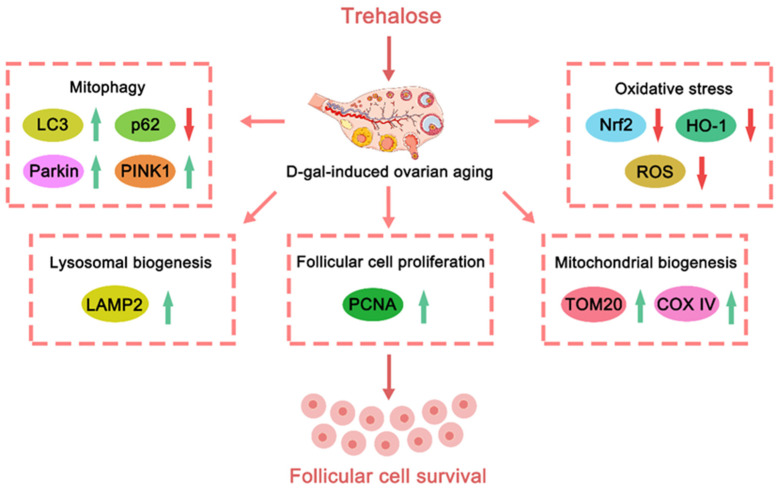
Schematic illustration of Tre attenuates D-gal-induced ovarian aging in mice.

In summary, the present study demonstrates that Tre attenuates ovarian aging in mice and inhibits D-gal-induced aging-related granulosa cell death at both tissue and cellular levels. Furthermore, Tre increases the expression levels of mitophagy-related proteins (Parkin, PINK1 and LC3), and improves mitophagy activity, thereby preventing oxidative stress and promoting mitochondrial biogenesis in granulosa cells (Figure 7). These findings contribute to the growing evidence for the potential of Tre as an anti-aging treatment for ovaries.

## Data Availability

All data generated or analyzed during this study are available from the corresponding author by request.

## Abbreviations

Tre: Trehalose; D-gal: D-galactose; ROS: reactive oxygen species; DAPI: 4′,6-diamidino-2-phenylindole; HO-1: Heme oxygenase-1; Nrf2: Nuclear factor erythroid 2-related factor 2; PINK1: PTEN-induced kinase 1; LC3: Microtubule-associated protein 1A/1B-light chain 3; COX IV: Cytochrome c oxidase polypeptide IV; PCNA: Proliferating cell nuclear antigen; p62: SQSTM1, sequestosome 1; LAMP2: Lysosome-associated membrane protein 2; TOM20: Translocase of outer mitochondrial membrane 20; ACTB: β-actin; DMEM/F12: Dulbecco’s modified eagle’s medium F-12; FBS: fetal bovine serum; SEM: standard error of the mean.
